# Modified Buckwheat Husk as a Filler for Urea–Formaldehyde Resin in Plywood Production

**DOI:** 10.3390/polym16101350

**Published:** 2024-05-10

**Authors:** Jakub Kawalerczyk, Joanna Walkiewicz, Ján Sedliačik, Dorota Dukarska, Magdalena Woźniak, Radosław Mirski

**Affiliations:** 1 Department of Mechanical Wood Technology, Faculty of Forestry and Wood Technology, Poznań University of Life Sciences, 60-627 Poznań, Poland; joanna.walkiewicz@up.poznan.pl (J.W.); dorota.dukarska@up.poznan.pl (D.D.); radoslaw.mirski@up.poznan.pl (R.M.); 2 Department of Furniture and Wood Products, Faculty of Wood Science and Technology, Technical University in Zvolen, 96053 Zvolen, Slovakia; sedliacik@tuzvo.sk; 3 Department of Chemistry, Faculty of Forestry and Wood Technology, Poznań University of Life Sciences, 60-627 Poznań, Poland; magdalena.wozniak@up.poznan.pl

**Keywords:** buckwheat husk, acetylation, silanization, filler, urea–formaldehyde adhesive, plywood

## Abstract

The aim of the presented research was to determine the suitability of both non-modified and modified buckwheat husk (BH) as a filler for urea–formaldehyde adhesive in plywood production. The effect of two modification methods, acetylation and silanization, was investigated. Infrared spectroscopy outcomes confirmed that both acetylation and silanization of the filler had occurred. Based on the results, it was found that the introduction of BH had a significant effect on both the adhesive properties and the characteristics of the manufactured plywood. The application of non-modified husks led to a reduction in viscosity and an extension of the gelation time, and the produced plywood boards were characterized by reduced bonding quality and increased delamination. Modification of the husk surface by acetylation and silanization with 3-aminopropyltriethoxysilane contributed to the noticeable improvement in the resin properties. On the other hand, the improvement in plywood properties, consisting of the increase in bonding quality and reduced delamination, was observed only in the case of the silanized husk. Furthermore, the use of non-modified and acetylated husk did not significantly influence the formaldehyde emission. The reduction in the investigated emission of formaldehyde was observed only in the case of variants containing 15 and 20% of silanized buckwheat husk.

## 1. Introduction

Plywood is a valuable wood-based material widely used in many industries, such as furniture, construction, packaging, etc. Even though it has been available on the market for so many years, its global production still reaches millions of cubic metres each year. According to Zheng et al. [1], in 2021, the worldwide production volume reached 482 million cubic metres. Such large production on a global scale also requires the synthesis of a significant amount of binding agents. Currently, approx. 95% of total adhesives applied in the production of wood-based panels are formaldehyde-based resins [2]. Urea–formaldehyde (UF) resin, the product of a reaction between urea and formaldehyde, is by far the most commonly used thermosetting adhesive for interior-grade plywood. Its annual global production may reach up to 11 million tons [3]. The reason for the wide use of UF resins on an industrial scale are, for example, good adhesion to wood, low curing temperature, ease of use, short pressing time, lack of colour, aqueous solubility and relatively low price [4,5]. In the case of plywood production, the filler is also an essential component of adhesive mixtures. Fillers are included mainly to adjust the viscosity of the adhesive. A higher viscosity facilitates the spreading of the adhesive on the surface of veneers and prevents overpenetration into their porous structure [6]. Moreover, replacing part of the synthetic resin by fillers can lead to a reduction in the overall production cost and the environmental impact of the manufactured material [7]. Furthermore, according to Cao et al. [6], the use of a properly selected filler can lead to an increase in the mechanical properties of the bond line, reduction in joint stress and improvement in the operation process.

In practice, various types of flour are most often added as fillers, mainly rye flour or wheat flour [8]. However, according to Liu et al. [9], the search for alternatives is gaining a lot of attention due to the waste of grain resources that could be used in the food industry. It is estimated that 1.5–2.0 million tons of flour were introduced as a filler in plywood production in 2021 [10]. Various types of waste biomass, which have not yet found a wider industrial application, may be considered as a promising alternative. A previously studied example is a biomass obtained from the forest and wood industry, such as bark [11], needles [12] or wood dust [13]. Furthermore, according to Hejna et al. [14], using lignocellulosic wastes from agricultural and food industries in the form of various husks, shells and leaves as the fillers for polymeric matrixes is an interesting concept that has recently been getting a lot of attention. In the case of UF adhesive-bonded plywood production, previously conducted research concerned, for example, chestnut shells [15], *Camellia oleifera* shells [10], scallop shells [16], walnut shells [17], pecan shells [18], macadamia shells [19], rice husks [20], etc. However, the results of the majority of these studies show that replacing flour with the ground lignocellulosic biomass usually contributes to the reduction in the strength characteristics of the produced plywood. Interestingly, according to Zwawi [21], chemical modification of bio-based fillers may contribute to the improvement in the materials’ performance; however, in the case of fillers for formaldehyde-based resin, the number of such studies is very limited. In most cases, coupling agents are used to modify the fillers. According to Mangore et al. [22], they can be defined as compounds applied to improve the chemical bonding between dissimilar materials and improve the interfacial bonding between the lignocellulosic filler and the polymer matrix. When it comes to this research, acetylation with sulfuric acid was chosen due to the overall simplicity of the method, while silanization using APTES was chosen because, according to Neves et al. [23], it is the most frequently used method of surface modification of cellulose-based fillers for the composites. An example of the bio-based material that has not yet been studied as the filler for UF resin in plywood production is buckwheat husk. Buckwheat belongs to the *Polygonaceae* family, and the two most cultivated species are *Fagopyrum tataricum* and *Fagopyrum esculentum* [24]. It is widely cultivated around the world mainly for food production due to its high content of proteins, amino acids, fibre, vitamins and minerals [25]. Moreover, according to Kuznetsova et al. [26], buckwheat husk is a source of flavonoids and phenolic acids, which, as shown by the outcomes of previous research on the use of phenolic-rich biomass, may be beneficial for the use as the UF resin filler. This type of husk was previously used as a filler for various polymeric materials, such as polyurethane-based composites [27], polypropylene-based composites [28], polyethylene-based composites [29] and poly(lactic acid)-based composites [30], and therefore, it can be an interesting choice for formaldehyde-based resin as well.

In summary, considering the constant search for new fillers from waste biomass for the production of plywood, the fact that buckwheat husk has not been used for this purpose so far and the promising results obtained with the addition of surface-modified fillers for polymeric matrixes, the aim of this research was to determine the effect of using modified buckwheat husk as a UF resin filler on the properties of the produced plywood.

## 2. Materials and Methods

### 2.1. Materials

The buckwheat husk (*Fagopyrum esculentum*) was purchased from Masala Deco company (Granica, Poland). Commercially available rye flour, containing 12.5% proteins, 70.4% carbohydrates, 1.5% ash and 1.5% fat, was used as a reference filler. Urea–formaldehyde resin with the following properties, a viscosity of 550 mPa·s, density of 1.28 g/cm^3^, solids content of 62.5%, pH of 7.5 and gel time at 100 °C for 140 s, was supplied by the local manufacturer of plywood. The plywood was produced using birch (*Betula* spp.) veneer with the dimensions of 320 × 320 mm, an average thickness of 1.4 mm, an average a density of 550 kg/m^3^ and moisture content of 4 ± 1%. All the reagents, such as 3-aminopropyltriethoxysilane (Merck, Poznań, Poland), acetic acid (99.9% pure, POCH, Gliwice, Poland), acetic anhydride (pure for analysis, Chempur, Piekary Śląskie, Poland), acetylacetone (pure for analysis, Chempur, Piekary Śląskie, Poland), ammonium acetate (pure for analysis, Chempur, Piekary Śląskie, Poland), ammonium nitrate (pure for analysis, Chempur, Piekary Śląskie, Poland), ethanol (pure for analysis, POCH, Gliwice, Poland), sodium hydroxide (pure for analysis, POCH, Gliwice, Poland) and sulphuric acid (pure for analysis, POCH, Gliwice, Poland), were used as received.

### 2.2. Buckwheat Husk Preparation and Modification

**Figure 1:**
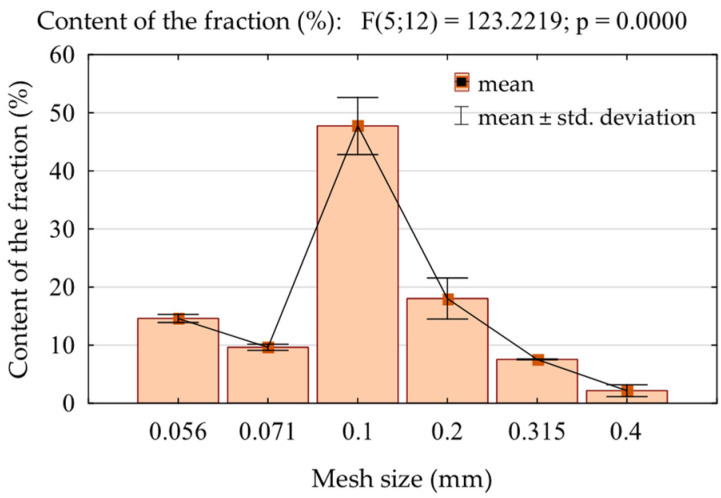
Fractional composition of ground buckwheat husk.

The buckwheat husk was ground using an IKA A10 basic laboratory mill (Staufen, Germany). To assess the particle size distribution, the obtained powder was passed through a set of sieves with the following mesh sizes: 0.056, 0.071, 0.100, 0.200, 0.315 and 0.400 mm. The determination of the fractional composition was repeated 3 times, and the average results are presented in Figure 1. Only fractions 0.2 mm and smaller were collected and used in this research. Then, the obtained powder was dried at 50 °C to reach a moisture content (MC) of 3 ± 1%.

**Figure 2:**
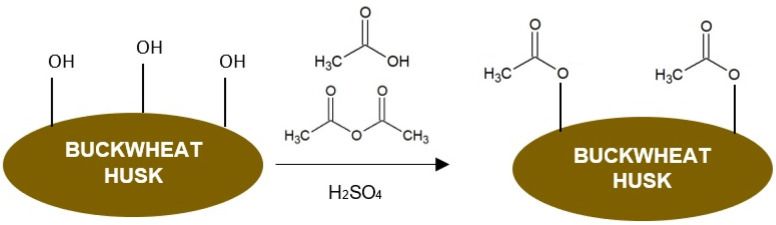
The schematic presentation of the buckwheat husk acetylation.

The first method of buckwheat husk modification was acetylation. This was performed by using the conditions described by Włoch and Landowska [27]. For this purpose, 50 g of husk powder was soaked in the mixture containing 2 mL of concentrated sulphuric acid, 198 mL of acetic acid and 200 mL of acetic anhydride. The modification was carried out for 48 h at room temperature while stirring at 400 rpm. The acetylated powder was washed with distilled water until neutral pH was reached and dried at 50 °C for 72 h. The schematic presentation of the performed acetylation is shown in Figure 2.

**Figure 3:**
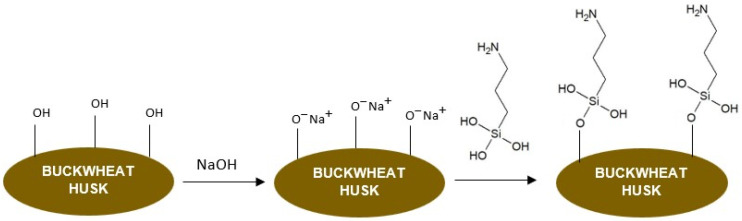
The schematic presentation of the buckwheat husk silanization.

The second method had two steps. The aim of the first step, which was mercerization in NaOH solution, was to activate the surface and increase the availability of hydroxyl groups. It was performed following the procedure described by Vázquez-Fletes et al. [29]. For this purpose, 50 g of ground husk was soaked in 500 mL of 5% NaOH solution at room temperature for 10 min while stirring at 400 rpm. Then, the suspension was filtered, washed with distilled water until neutral pH was obtained and dried at 80 °C for 12 h. The second step was silanization with 3-aminopropyltriethoxysilane (APTES) using the method described by Woźniak and Ratajczak [31] with a slight modification. The concentration of APTES in the solvent (a mixture of ethanol and water in a mass ratio of 80:20) was 20%. The mixture was stirred for 30 min and then the ground husk particles were soaked in the solution in a 1:5 mass ratio. The pH of the solution was decreased to 4 with acetic acid to increase the silanization effectiveness [32]. The reaction was run at room temperature for 3 h while stirring at 400 rpm. Then, the suspension was filtered, washed with distilled water to reach a neutral pH and dried at 50 °C for 48 h. The schematic presentation of the performed silanization is shown in Figure 3.

The effectiveness of the performed modifications was assessed with the attenuated total reflectance–Fourier transform infrared spectroscopy (ATR-FTIR) using a Nicolet iS5 spectrophotometer (Thermo Fisher Scientific, Waltham, MA, USA) with a deuterated triglycine sulfate detector.

### 2.3. Plywood Manufacturing and Testing

The variants used in this research were designed to differ in both the amount and type of filler. The compositions of the individual adhesive mixtures are presented in Table 1. In addition to the filler and resin, the mixtures also contained 3% of the 20% ammonium nitrate solution as the hardener. After its addition, the components were stirred manually until a homogeneous mixture was obtained.

In order to investigate the effect of the assumed variables on the characteristics of the mixtures, the parameters commonly used to control the quality of resins were analysed in triplicate. The viscosity was determined with the use of a rotary viscometer Brookfield DV-II+Pro (Middleboro, MA, USA). The gel time was determined according to Polish standard PN-C-89352-3 [33]. The pH was measured using a Testo 206 pH-meter (Pruszków, Poland).

The adhesive mixtures were spread on the surface of the external veneers in the amount of 150 g/m^2^. The three-layer sets were hot pressed at 120 °C for 4 min with a unit pressure of 1.4 MPa to produce three sheets of plywood for each variant. To determine the adhesive behaviour in the manufactured plywood, the bonding quality (f_v_) after soaking in water for 24 h was determined according to EN 314-1 [34], using 10 samples from each variant. The strength of the bond lines in changing conditions causing internal stresses is also an important parameter. Therefore, the tendency of plywood to delaminate was examined in accordance with ANSI/HPVA HP-1 [35] after one cycle of treatment, including soaking in water at 24 ± 2 °C for 4 h followed by drying in a laboratory oven for 19 h at 50 ± 1 °C. According to the standard, delamination is a continuous opening between two veneer layers deeper than 6.35 mm, longer than 50 mm and wider than 0.08 mm. The test was performed using 10 samples of each variant. The mechanical properties such as the bending strength and modulus of elasticity were determined according to EN 310 [36] in both the parallel and perpendicular directions to the grain direction of the wood using 12 samples from each variant. The formaldehyde emission was analysed in triplicate using the flask method according to EN 717-3 [37]. The content of the formaldehyde in the collected water solution was measured using the ammonium acetate and acetylacetone method with a Biosens UV-5600 spectrophotometer (Warsaw, Poland) at a wavelength of 412 nm.

### 2.4. Statistical Analysis

In order to analyse the obtained results, the two-factor analysis of variance (ANOVA) was performed with the use of Statistica 13.0 software. To assess the statistical significance of the observed differences between the variants, the HSD Tukey test was used at the significance level of α = 0.05.

## 3. Results and Discussion

**Figure 4:**
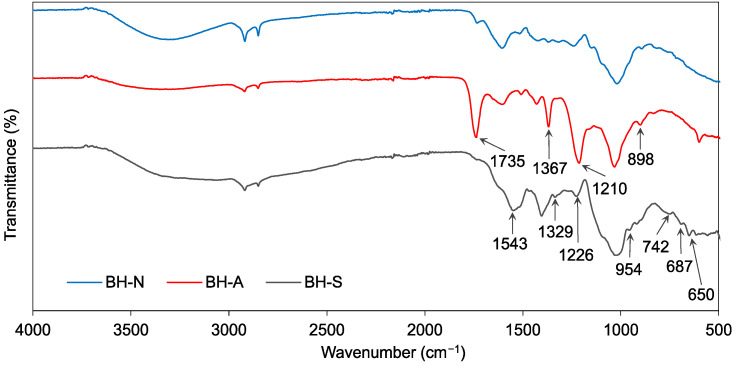
IR spectra of buckwheat husk.

The IR spectra of the raw buckwheat husk (BH-N) and after modification, namely, acetylation (BH-A) and silanization (BH-S), are presented in Figure 4. In the spectrum of the acetylated buckwheat husk, the increase in the transmittance intensity of the bands at 1735 cm^−1^ (C=O stretching), 1367 cm^−1^ (methyl vibration), 1210 cm^−1^ (C–O stretching) and 898 cm^−1^ (methyl vibration), compared to the transmittance intensity in the spectrum of the non-modified filler, indicated a successful acetylation process [27,38]. The spectrum of the silanized buckwheat husk shows bands confirming the silanization process with APTES, including the band at 1543 cm^−1^ corresponding to the N-H in-plane bending and the bands at 1329 cm^−1^ and 1226 cm^−1^ corresponding to the C-N stretching [39,40]. This spectrum also included bands at 687 cm^−1^ and 650 cm^−1^, characteristic of the vibrations of the Si–C and Si–O groups, respectively, originating from the ethoxy group in (3-aminopropyl)triethoxysilane [40,41]. Additionally, the bands at 954 cm^−1^ and 742 cm^−1^ can also be attributed to the vibrations of Si-C and/or Si-O in triethoxysilyl groups [42,43].

The outcomes presented in Table 2 show that both the type of filler and its share had a significant effect on the properties of the adhesive mixtures, such as the viscosity and gel time. On the other hand, the introduction of the buckwheat husk powder, regardless of its loading and modification, did not affect the pH of the mixture. It was found that the mixtures containing rye flour were characterized by a significantly higher viscosity compared to those containing buckwheat husks. Flour, demonstrating the ability to absorb a significant amount of water from the mixture, undergoes gelatinization at high temperature. As a result, a colloidal gelling structure is created, which promotes the increase in viscosity to the level that is not usually achievable for waste biomass at the same loading [8]. The filler loading also had a significant effect on the viscosity. As expected, as the share of the ground husks increased, the viscosity values also increased. Furthermore, both the acetylation and silanization resulted in an increase in viscosity compared to the variants containing the non-modified husk. Both modification methods can result in a more uniform dispersion of lignocellulosic fillers in the polymer matrix [44,45], which consequently, according to Guchait et al. [46], may contribute to the increase in the viscosity of adhesive mixtures. Moreover, the type of filler significantly influenced the gel time of the mixtures as well. The results have shown that the gel time of the mixtures containing flour was considerably shorter than in the case of the mixtures containing ground husks. The ongoing cross-linking of the network created by the proteins of the flour probably improved the reactivity and accelerated the gel time [47]. The acceleration of the gel time was also observed with the increasing loading of the husk powder due to the more intense water absorption, which may favour the curing process. What is also interesting is that it was found that the modification of the husks with the coupling agents also contributed to the acceleration of the gel time. The reason was probably a combination of better filler dispersion and the enhanced compatibilization between the lignocellulosic filler and the synthetic polymer, which was previously described in the case of both acetylated and silanized fillers for composites [48,49].

The results of the ANOVA are presented in Table 3 and confirm the significant effect of the share of the filler and its modification on the gel time and viscosity of the investigated adhesive formulations. The interaction of these factors was also significant. This is confirmed by the high values of the sum of squares (SS), mean square (MS) and Fisher statistic (F) and the *p*-value below 0.05. However, the analysed factors did not have a significant effect on the pH of the adhesive mixtures. In this case, the statistical parameters such as the SS and MS reached the level of 0, and the *p*-value significantly exceeded the assumed level of significance.

**Figure 5:**
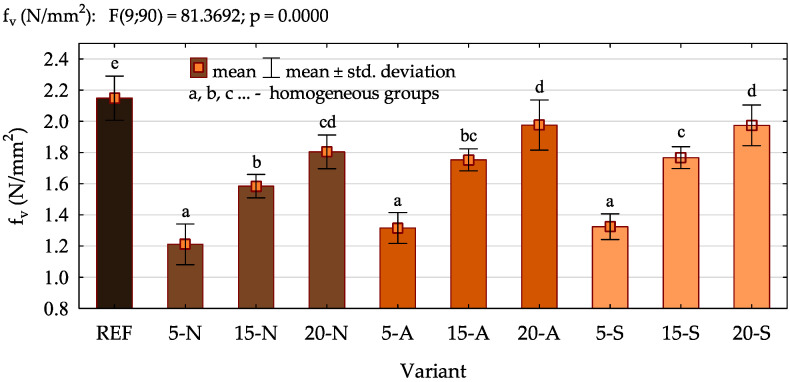
The results of the bonding quality test.

The results of the bonding quality determined in the wet shear strength test (f_v_), which is considered to be the basic indicator of the adhesive behaviour in plywood, are presented in Figure 5. Based on the outcomes, it was found that the reference plywood was characterized by the highest strength of the bond lines. The progressing gelatinization of flour at high temperature during pressing leads to the creation of a cross-linked structure, providing high homogeneity of the cured adhesive. In the case of biomass particles, it may be difficult to achieve, especially considering the tendency of fine particles to agglomerate. According to Gao et al. [50], the formation of agglomerates may lead to the deterioration of the morphology of the cured resin by the formation of microcracks and void spaces. Moreover, their formation also hinders the transfer of stresses in the bond line by concentrating them at certain points [51]. Markedly, it was also noticed that as the loading of the buckwheat husk increased, the shear strength values also increased. The reason was most likely the adjustment of the viscosity, which prevented the resin from penetrating the veneer, and consequently, the amount remaining on the veneer surface allowed for the creation of a bond line characterized by good quality [6]. Furthermore, the analysis of the homogeneous groups showed that the filler modification resulted in considerably less noticeable changes. No significant effect of filler acetylation was observed. However, it seems that the silanization contributed to the improvement in the bonding quality. The comparison of the variants containing 15% of the ground husk showed a 10% increase in strength as a result of the husk’s modification with APTES. This positive effect was most likely caused by the increased reactivity of the adhesive, improved compatibilization between the adhesive and filler and better dispersion of the filler within the adhesive. The enhancement in UF resin performance due to the filler modification using APTES was also noticed in the case of research on, for example, ground needles [12], nanocellulose [52] and nanoclay [53].

The delamination test is also a valuable indicator of the bonding quality in plywood [54]. In most cases, delamination is caused by the hydrolysis of bonds during soaking and the internal stresses among the adjacent layers created due to shrinking and swelling in changing conditions. Delamination usually leads to buckling and rapid deterioration in mechanical properties due to the loss of rigidity [55,56]. Similarly, as in the case of wet shear strength, the reference variant of the plywood demonstrated the lowest susceptibility to delamination, lower than the variants containing the non-modified husk (Table 4). Moreover, a positive effect of the increase in the loading of the husk was observed as well. Interestingly, the results also indicate a positive effect of both modification methods. The use of acetylated and silanized buckwheat husk powder in amounts of 15 and 20% resulted in no delamination, as in the case of the reference variant. The lack of delaminated samples is crucial because, according to the American National standard for hardwood and decorative plywood [35], a manufactured panel passes the delamination test only if 95% of samples do not delaminate after the first cycle of pretreatment.

The results of the mechanical properties determined both in the perpendicular and parallel directions are summarized in Table 5. It was found that that the only statistical differences were observed in the case of the variants containing 5% of the buckwheat husk, both modified and unmodified. It was most likely caused by a too low viscosity, which significantly reduced the strength of the bond lines and, consequently, also the mechanical properties of the plywood. The lack of influence of the other variables related to the adhesive formulation probably results from the fact that both the bending strength and elastic modulus of plywood depend mainly on the properties of the veneers. According to Bal and Bektaş [57], among these properties, the density of the veneer is crucial, but the effect of the species, number of plies, thickness and drying temperature can also be observed. Therefore, it seems that only in the case of variants with the lowest filler loading was the reduction in the strength of bond lines significant enough to affect the results of the mechanical properties in a statistically significant way.

**Figure 6:**
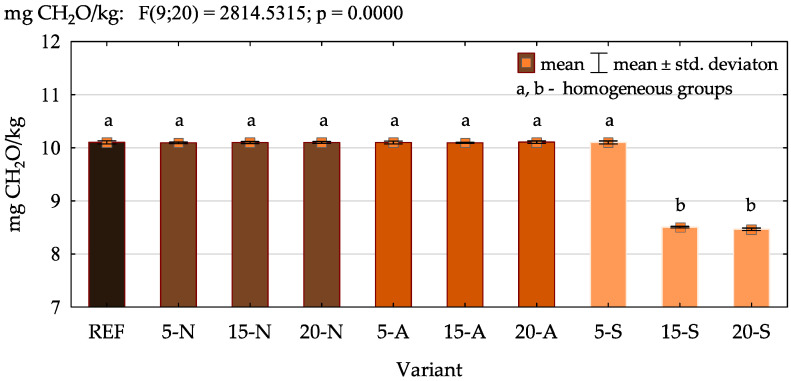
The results of formaldehyde emission.

Considering that the long-term exposure to indoor formaldehyde release can pose a high risk to human health, the limits of permissible emissions are constantly becoming more stringent. Therefore, it is important to find fillers that will allow for the production of plywood characterized by a lower formaldehyde release [58,59]. The results of formaldehyde emission are shown in Figure 6. Although buckwheat husk contains bioactive compounds such as phenolic acids and flavonoids, no statistically significant changes were observed when non-modified or acetylated husks were used, regardless of their amount. The only significant differences were noticed in the case of the variants containing 15 and 20% of the silanized husk. In their case, the amount of released formaldehyde was reduced by approx. 16%. The reason was probably the amino group located on top of the APTES molecule. According to Hassannejad et al. [60], it is the most effective reactive group for formaldehyde adsorption, and therefore, it can react with both free and hydrolyzed formaldehyde in UF resin-bonded plywood [61].

The results of the ANOVA presented in Table 6 confirmed a significant effect of the amount of filler introduced to the UF adhesive on every tested property of plywood. Based on the high values of the SS, MS and F and a *p*-value of 0, it was found that the choice of appropriate loading can contribute to the improvement in the bonding quality, flexural characteristics and reduction in formaldehyde emission. Furthermore, it was confirmed that the positive effect of the filler modification was observed in the case of the results of the bonding quality and formaldehyde emission. Interestingly, the interaction between the analysed variables (the amount of filler and the method of its modification), indicating the occurrence of a synergistic effect, was observed only in the case of the formaldehyde release.

## 4. Conclusions

Based on the conducted research, it was found that the use of buckwheat husk as a filler for UF resin had a significant effect on both the adhesive properties and the characteristics of manufactured plywood. The addition of husk powder did not significantly affect the pH of the mixture; however, it reduced its viscosity and extended the gel time. Moreover, the modification of the filler also had an effect on the properties of the adhesive. Both acetylation and silanization led to an increase in viscosity and shortening of the gel time compared to the non-modified filler, but the results were still slightly worse than in the case of the reference mixture. The use of buckwheat husks led to a decrease in the bonding quality of the plywood and the increase in its tendency to delamination. The worst results were observed in the variants containing 5% ground husk, probably due to unsuitable viscosity. However, the results improved with the increase in the loading of the filler. Among the modification methods used, the silanization turned out to be particularly effective because it led to the increase in the bonding quality and the lack of delamination. The results also showed that mechanical properties depended mainly on the amount of filler, and the statistically significant reduction was observed only in the case of variants containing the lowest filler content. Furthermore, the introduction of non-modified and acetylated husk did not significantly affect the formaldehyde emission from the plywood. A reduction in formaldehyde emissions was observed only in the case of the variants containing 15 and 20% of silanized buckwheat husk.

## Figures and Tables

**Table 1:** Composition of adhesive mixtures.

Variant Label	Type of Filler	Share of Filler (%)
REF	Flour	15
5-N	Non-modified husk	5
15-N	Non-modified husk	15
20-N	Non-modified husk	20
5-A	Acetylated husk	5
15-A	Acetylated husk	15
20-A	Acetylated husk	20
5-S	Silanized husk	5
15-S	Silanized husk	15
20-S	Silanized husk	20

**Table 2:** Properties of adhesive mixtures.

Variant Label	Viscosity (mPa·s)	Gel Time (s)	pH
REF	3090 ± 23 ^f^	267 ± 6 ^a^	6.71 ± 0.01 ^a^
5-N	1196 ± 13 ^a^	374 ± 5 ^f^	6.70 ± 0.02 ^a^
15-N	2279 ± 27 ^b^	311 ± 4 ^d^	6.69 ± 0.02 ^a^
20-N	2659 ± 21 ^d^	299 ± 3 ^cd^	6.70 ± 0.01 ^a^
5-A	1231 ± 18 ^a^	343 ± 3 ^e^	6.71 ± 0.02 ^a^
15-A	2492 ± 31 ^c^	300 ± 2 ^cd^	6.70 ± 0.02 ^a^
20-A	2883 ± 15 ^e^	284 ± 4 ^b^	6.70 ± 0.02 ^a^
5-S	1231 ± 11 ^a^	341 ± 2 ^e^	6.69 ± 0.01 ^a^
15-S	2492 ± 21 ^c^	298 ± 8 ^c^	6.69 ± 0.02 ^a^
20-S	2895 ± 14 ^e^	282 ± 4 ^b^	6.70 ± 0.01 ^a^

The values denoted with identical letters do not differ significantly at *p* = 0.05 according to the post hoc test, following the ANOVA test.

**Table 3:** The results of the ANOVA performed on the results of the properties of the adhesive mixtures.

Main Factor	Statistical Parameters				
	*SS*	*Df*	*MS*	*F*	*p*
Gel time (s)					
A	20,404	2	10,202	606.7	0.0000
B	2380	2	1190	70.8	0.0000
A × B	459	4	115	6.8	0.0016
pH					
A	0.000	2	0.000	1	0.3994
B	0.000	2	0.000	1	0.5284
A × B	0.000	4	0.000	0	0.7479
Viscosity (mPa·s)					
A	12,406,487	2	6,203,243	16,795.8	0.0000
B	151,868	2	75,934	205.6	0.0000
A × B	47,079	4	11,770	31.9	0.0000

A—share of filler, B—modification method, A × B—interaction between main factors, SS—sum of squares, Df—degrees of freedom, MS—mean square and F—Fisher statistic, *p*-value.

**Table 4:** The results of the delamination test.

Variant Label	Number of Delaminated Samples
REF	0/10
5-N	5/10
15-N	2/10
20-N	1/10
5-A	4/10
15-A	0/10
20-A	0/10
5-S	4/10
15-S	0/10
20-S	0/10

**Table 5:** The results of the mechanical properties.

Variant Label	Bending Strength (N/mm^2^)		Modulus of Elasticity (N/mm^2^)	
	⊥	||	⊥	||
REF	32.1 ± 1.8 ^b^	135.5 ± 5.5 ^b^	1381.5 ± 29.8 ^b^	14,230 ± 292 ^b^
5-N	19.0 ± 0.7 ^a^	107.2 ± 8.2 ^a^	1110.2 ± 95.4 ^a^	10,889 ± 516 ^a^
15-N	31.4 ± 1.4 ^b^	134.4 ± 8.1 ^b^	1386.0 ± 32.4 ^b^	14,182 ± 306 ^b^
20-N	31.3 ± 1.5 ^b^	136.5 ± 7.0 ^b^	1384.0 ± 41.3 ^b^	14,141 ± 271 ^b^
5-A	19.1 ± 0.9 ^a^	107.6 ± 7.7 ^a^	1115.8 ± 78.7 ^a^	10,904 ± 869 ^a^
15-A	31.7 ± 1.4 ^b^	136.6 ± 4.2 ^b^	1381.3 ± 46.6 ^b^	14,211 ± 551 ^b^
20-A	32.0 ± 1.2 ^b^	136.5 ± 6.1 ^b^	1384.7 ± 40.7 ^b^	14,189 ± 310 ^b^
5-S	19.2 ± 0.8 ^a^	107.0 ± 7.8 ^a^	1110.7 ± 74.6 ^a^	10,806 ± 874 ^a^
15-S	31.5 ± 0.7 ^b^	136.5 ± 5.3 ^b^	1380.8 ± 54.1 ^b^	14,164 ± 486 ^b^
20-S	31.6 ± 1.2 ^b^	136.4 ± 8.0 ^b^	1384.2 ± 52.6 ^b^	14,171 ± 379 ^b^

⊥—perpendicular to the grain direction of the wood and ||—parallel to the grain direction of the wood. The values denoted with identical letters do not differ significantly at *p* = 0.05 according to the post hoc test, following the ANOVA test.

**Table 6:** The results of the ANOVA performed on the results of the plywood properties.

Main Factor	Statistical Parameters				
	*SS*	*Df*	*MS*	*F*	*p*
f_v_ (N/mm^2^)					
A	6.3121	2	3.1561	271.06	0.0000
B	0.4173	2	0.2087	17.92	0.0000
A × B	0.0132	4	0.0033	0.28	0.8886
MOR ⊥ (N/mm^2^)					
A	10,023.6	2	5011.8	100.57	0.0000
B	4.6	2	2.3	0.05	0.9545
A × B	17.2	4	4.3	0.09	0.9863
MOR || (N/mm^2^)					
A	1865.29	2	932.65	757.56	0.0000
B	1.03	2	0.52	0.42	0.6595
A × B	0.47	4	0.12	0.10	0.9835
MOE ⊥ (N/mm^2^)					
A	880,752	2	440,376	119.65	0.0000
B	40	2	20	0.01	0.9946
A × B	161	4	40	0.01	0.9998
MOE || (N/mm^2^)					
A	1.3 × 10^8^	2	6.6 × 10^7^	216.37	0.0000
B	2.7 × 10^4^	2	1.3 × 10^4^	0.04	0.9565
A × B	3.3 × 10^4^	4	8.4 × 10^3^	0.03	0.9985
Formaldehyde emission (mg CH_2_O/kg)					
A	1.728	2	0.864	1944	0.0000
B	6.912	2	3.456	7776	0.0000
A × B	3.502	4	0.875	1970	0.0000

A—share of filler, B—modification method, A × B—interaction between main factors, SS—sum of squares, Df—degrees of freedom, MS—mean square and F—Fisher statistic, *p*-value.

## Data Availability

The original contributions presented in the study are included in the article, further inquiries can be directed to the corresponding author.
